# Hippocampal activation correlates with visual confrontation naming: fMRI findings in controls and patients with temporal lobe epilepsy

**DOI:** 10.1016/j.eplepsyres.2011.04.007

**Published:** 2011-08

**Authors:** Silvia B. Bonelli, Rob Powell, Pamela J. Thompson, Mahinda Yogarajah, Niels K. Focke, Jason Stretton, Christian Vollmar, Mark R. Symms, Cathy J. Price, John S. Duncan, Matthias J. Koepp

**Affiliations:** a Epilepsy Society MRI Unit, Department of Clinical and Experimental Epilepsy, UCL Institute of Neurology, Queen Square, London WC1N 3BG, UK; b Department of Clinical Neurophysiology, Georg-August University, 37099 Goettingen, Germany; c Functional Imaging Laboratory, Wellcome Centre for Imaging Neuroscience, University College London, London WC1N 3BG, UK

**Keywords:** Temporal lobe epilepsy, fMRI, Language, Naming, Hippocampus

## Abstract

**Purpose:**

In patients with left temporal lobe epilepsy (TLE) due to hippocampal sclerosis (HS) decreased naming ability is common, suggesting a critical role for the medial left temporal lobe in this task. We investigated the integrity of language networks with functional MRI (fMRI) in controls and TLE patients.

**Experimental design:**

We performed an fMRI verbal fluency paradigm in 22 controls and 66 patients with unilateral mesial TLE (37 left HS, 29 right HS). Verbal fluency and naming ability were investigated as part of the standard presurgical neuropsychological assessment. Naming ability was assessed using a visual confrontation naming test.

**Results:**

Left TLE patients had significantly lower naming scores than controls and those with right TLE. Right TLE patients performed less well than controls, but better than those with left TLE. Left TLE had significantly lower scores for verbal fluency than controls.

In controls and right TLE, left hippocampal activation during the verbal fluency task was significantly correlated with naming, characterised by higher scores in subjects with greater hippocampal fMRI activation. In left TLE no correlation with naming scores was seen in the left hippocampus, but there was a significant correlation in the left middle and inferior frontal gyri, not observed in controls and right TLE. In left and right TLE, out of scanner verbal fluency scores significantly correlated with fMRI activation for verbal fluency in the left middle and inferior frontal gyri.

**Conclusion:**

Good confrontation naming ability depends on the integrity of the hippocampus and the connecting fronto-temporal networks. Functional MRI activation in the left hippocampus during verbal fluency is associated with naming function in healthy controls and patients with right TLE. In left TLE, there was evidence of involvement of the left frontal lobe when naming was more proficient, most likely reflecting a compensatory response due to the ongoing epileptic activity and/or underlying pathology.

## Introduction

Temporal lobe epilepsy (TLE) is commonly associated with impairment in cognitive function, in particular memory and language function. The impairment of episodic memory is well recognised and the medial temporal lobe and in particular the hippocampus has been shown to be crucial for memory encoding and retrieval (Squire and Zola-Morgan, 1991). The impairment of language function in patients with TLE in contrast is less well understood. Up to 40% of patients with TLE and a speech dominant focus have a clinically significant deficit in naming abilities (Bell et al., 2003; Loring et al., 1994) which is often aggravated following anterior temporal lobe resection (ATLR) of the language dominant hemisphere (Davies et al., 1998).

Functional MRI is an attractive clinical tool to evaluate cognitive function as it is non-invasive and highly reproducible in particular for localising the parts of the brain involved in processing language function (Rutten et al., 2002a,b). FMRI studies using simple phonemic verbal fluency paradigms reliably lateralise language dominance in healthy controls and TLE patients by showing left frontal lobe activation corresponding to Broca's area, and less prominent activation in the medial temporal lobes (Friedman et al., 1998; Phelps et al., 1997).

It is well established that naming function is mediated by the perisylvian cortex in the language dominant hemisphere. More recently, there is accumulating evidence from cortical stimulation studies (Hamberger et al., 2007) as well as fMRI studies (Tomaszewki Farias et al., 2005) that the hippocampus is directly involved in naming functions.

ATLR in the language dominant hemisphere, a well established and effective treatment for patients with medically refractory TLE (Wiebe et al., 2001) carries the risk of postoperative naming deficits. About 25% of patients with TLE due to hippocampal sclerosis suffer a clinically significant naming decline after left ATLR (Davies et al., 1998; Saykin et al., 1995). Baseline naming has been reported to be poorer in patients with hippocampal sclerosis compared to patients without hippocampal sclerosis (Bell and Davies, 1998). However, the underlying mechanisms are still poorly understood. Bell and colleagues for example suggested that naming deficits in patients with left TLE were more attributable to a related semantic memory impairment rather than simply to retrieval deficits (Bell et al., 2001). Other studies discussed that naming difficulties in TLE are more likely to be due to lexical retrieval problems associated with the temporal lobe network (Trebuchon-Da Fonseca et al., 2009).

In this study, we retrospectively investigated the relationship between naming ability and the integrity of language networks using fMRI and a simple verbal fluency task. We tested the hypotheses that

1. Stronger fMRI activations in the lateral frontal and medial temporal lobes are associated with better verbal fluency and naming ability in healthy controls and patients with TLE.
2. There will be reorganisation of language function in patients with TLE in the speech-dominant hemisphere.

## Patients and methods

### Subjects

We studied 22 healthy controls (median age 42.50 years, range 22–70, 11 females) with no history of neurological or psychiatric disease and 66 patients undergoing presurgical evaluation at the National Hospital for Neurology and Neurosurgery for medically refractory TLE. All patients had undergone structural MRI at 3.0 T, showing left hippocampal sclerosis (HS) in 37 patients (median age 42 years, range 17–63, median age of epilepsy onset 7 years, range 0.50–44, 20 females) and right HS in 29 patients (median age 37 years, range 22–54, median age of epilepsy onset 10 years, range 0.75–25, 17 females). Prolonged interictal and ictal video-EEG monitoring confirmed that seizures were arising from the ipsilateral mesial temporal lobe (MTL) in all patients. On quantitative assessment all patients had normal contralateral MTL structures, in particular a normal hippocampus (Woermann et al., 1998). All patients were on antiepileptic medication at the time of their assessment with five left TLE and three right TLE patients receiving Topiramate, which has been associated with neuropsychological impairment such as slowed processing speed, especially in tests requiring verbal processing (Lee et al., 2003; Loring et al., 2011; Thompson et al., 2000). English was the first language of all subjects, handedness was assessed using the Edinburgh Hand Preference Inventory (Oldfield, 1971) standardised questionnaire. Language dominance was assessed calculating a lateralisation index (LI) using the Bootstrap method of the SPM toolbox (Wilke and Lidzba, 2007) for the contrast “verbal fluency” for each subject in the relevant regions of interest, the middle and inferior frontal gyri. Left language dominance was defined by a LI of ≤−0.2. IQ was measured using WAIS-III. All patients and controls were left hemisphere dominant for language. The mean verbal IQ in controls was 105.1 (SD 12.16), 97.07 (SD 16.72) in right TLE and 93.30 (SD 12.95) in left TLE patients; there was a significant difference in verbal IQ between controls and left TLE patients (ANOVA *p* < 0.01); the mean performance IQ in controls was 106.4 (SD 13.46), 95.59 (SD 15.66) in right TLE and 93.38 (SD 13.41) in left TLE patients (ANOVA *p* < 0.05).

The study was approved by the National Hospital for Neurology and Neurosurgery and the Institute of Neurology Joint Research Ethics Committee and informed written consent was obtained from all subjects.

### Naming

All patients and controls completed the Mc Kenna Graded Naming Test.

The subject is required to name 30 black and white line drawings of increasing difficulty. The total number of items correctly named is the performance indicator (McKenna and Warrington, 1983).

### Verbal fluency

All patients and controls completed a verbal fluency test outside the scanner.

The subject is given 60 s to produce as many words starting with a given letter (“S”). This is a non-standardised test used commonly in our clinical practise. The total number of words correctly produced is the performance indicator.

### MR acquisition

MRI studies were performed on a 3.0 T General Electric Excite HD scanner. Standard imaging gradients with a maximum strength of 40 mTm^−1^ and slew rate 150 Tm^−1^ s^−1^ were used. All data were acquired using an eight-channel array head coil for reception and the body coil for transmission.

For the fMRI task, gradient-echo planar T2*-weighted images were acquired, providing blood oxygenation level-dependent (BOLD) contrast. Each volume comprised 58 contiguous 2.5-mm oblique axial slices, in the plane of the long axis of the hippocampus, with a 24 cm field of view, 96 × 96 matrix, reconstructed to 128 × 128 for an in-plane resolution of 1.88 mm × 1.88 mm. Echo time (TE) was 30 ms, and repetition time (TR) was 4.5 s. The field of view was positioned to maximize coverage of the frontal and temporal lobes.

### Language fMRI task and data analysis

Each subject performed a simple verbal fluency fMRI task, which is known to reliably lateralise language (Powell et al., 2006). In brief, this paradigm consisted of a blocked experimental design with 30 s activation blocks alternating with 30 s of cross-hair fixation during the baseline condition over 5.5 min. During the activation phase, subjects were asked to covertly generate different words beginning with a visually presented letter (A, S, W, D and E).

The data were analysed using statistical parametric mapping (SPM5) (Friston et al., 1995) (Wellcome Trust Centre for Imaging Neuroscience (http://www.fil.ion.ucl.ac.uk/spm/)). Scans from each subject were realigned using the mean image as a reference, spatially normalized into standard space (using a scanner specific template created from 30 healthy controls, 15 patients with left HS and 15 patients with right HS) and spatially smoothed with a Gaussian kernel of 10 mm FWHM. A two level random effects analysis was employed.

At the first level, condition-specific effects were estimated according to the general linear model (Friston et al., 1995) for each subject and regressors of interest were formed by creating boxcar functions of task against rest. Parameter estimates for these regressors were then calculated for each voxel. One contrast image was produced for each subject within the three groups (controls, left and right TLE patients), corresponding to the main effects of verbal fluency against fixation.

The contrast image (verbal fluency relative to baseline) for each subject was then entered into a second level ANOVA, which modelled the group effect (i.e. control subjects or patients) on the contrast of interest. Naming and verbal fluency scores were entered as covariates separately for control subjects and patients. Inferences were made at the second level to emulate a random effects analysis and enable inferences at the population level (Friston et al., 1999). Given that our design was balanced, this two-stage procedure is mathematically identical to a random/mixed effects analysis. Verbal IQ was entered as an additional covariate of no interest to control for effect of variation in this measure.

At the second level, we tested for

1. Verbal fluency effects in control subjects.
2. Verbal fluency effects in patients.
3. Verbal fluency effects that differed between controls and patients.
4. Verbal fluency effects that increased with naming and verbal fluency in controls.
5. Verbal fluency effects that increased with naming and verbal fluency in patients.

Unless otherwise stated, we report all activations at a threshold of *p* < 0.05, corrected for multiple comparisons (family wise error rate (FWE)). For the correlational analysis we report all mesial temporal and frontal activations at a threshold of *p* < 0.01, corrected for multiple comparisons in a small volume of interest (SVI). In view of our hypothesis we performed the small volume correction using a sphere of 10 mm diameter for the left (and right) hippocampi and a sphere of 20 mm diameter for the middle and inferior frontal gyri based on the peak activation. MTL activations were labelled with reference to Duvernoy's the Human Hippocampus (Duvernoy, 1998).

## Results

### Naming test scores

There was a significant difference in naming scores between controls (mean 22.59, SD 3.45), left (mean 15.05, SD 3.94) and right TLE patients (mean 17.93, SD 4.94), with patients showing significantly lower scores than controls (ANOVA left: *p* < 0.001; right: *p* < 0.001, Bonferroni corrected for multiple comparisons) and left TLE patients demonstrating significantly lower naming scores than right TLE patients (ANOVA *p* < 0.01, Bonferroni corrected for multiple comparisons).

There was no significant correlation between age of epilepsy onset and naming test scores in left or right TLE patients.

### Verbal fluency test scores

Left TLE patients (mean 13.32, SD 5.57) demonstrated significantly lower scores for verbal fluency than controls (mean 18.41, SD 5.69) (ANOVA *p* < 0.01, Bonferroni corrected for multiple comparisons); there was no significant difference in verbal fluency scores between controls and right TLE patients (mean 15.00, SD 6.71) or left and right TLE patients.

There was no significant correlation between naming and verbal fluency scores outside the scanner.

### Hippocampal volumes

Left and right hippocampal volumes were significantly different in both left and right TLE patients:

Left TLE group: mean (SD) right hippocampal volume, 2.78 (0.28) cm^3^; mean left hippocampal volume, 1.72 (0.44) cm^3^, (paired *t*-test *p* < 0.0001, 2-tailed).
Right TLE group: mean (SD) right hippocampal volume, 1.75 (0.43) cm^3^; mean left hippocampal volume, 2.66 (0.33) cm^3^, (paired *t*-test *p* < 0.0001, 2-tailed).

There was no significant difference between left hippocampal volume in the left TLE group and right hippocampal volume in the right TLE group or between left and right hippocampal volume in controls. Controls’ hippocampal volumes did not differ significantly from contralateral hippocampal volumes in right and left TLE patients. No significant correlation was seen between left hippocampal volumes and naming scores in healthy controls and patients with left or right HS.

### fMRI results

Main effects of verbal fluency within each and across the three groups (Table 1).

**Figure 1:**
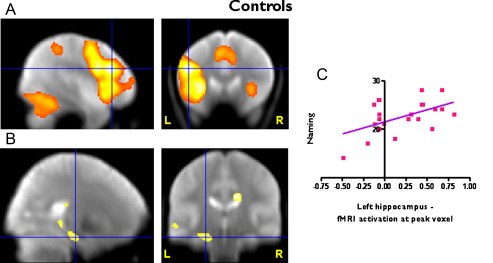
FMRI results in controls. (A) Main effect for verbal fluency, Left middle and inferior frontal activation (Threshold *p* < 0.05, FWE correction). (B) Correlational analysis. Left hippocampal activation for verbal fluency correlates with naming scores, characterised by better naming scores in subjects with greater fMRI activation (Threshold *p* < 0.01, uncorrected). (C) Correlation of fMRI activation for verbal fluency and naming scores at the peak voxel in the left hippocampus. Significant regions are superimposed onto an averaged normalized mean EPI image from 30 healthy controls, 15 patients with left and 15 patients with right HS.

Significantly left lateralised activation was demonstrated in the left middle (*p* < 0.0001, FWE corrected) and inferior (*p* < 0.0001, FWE corrected) frontal gyri for healthy controls (Fig. 1A) and patients with left and right HS. Group comparison revealed greater left frontal activation in controls compared to left (*Z* score = 4.19, *p* = <0.0001, uncorrected) and right TLE patients (*Z* score = 4.48, *p* < 0.0001, uncorrected). There was no significant difference between left and right TLE patients in left frontal activation.  

**Figure 2:**
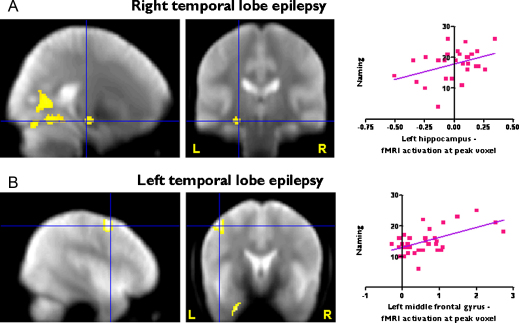
FMRI results in TLE patients. (A) Right TLE patients: correlational analysis. Left hippocampal activation for verbal fluency correlates with naming scores, characterised by better naming scores in subjects with greater fMRI activation (Display at threshold *p* < 0.01, uncorrected). (B) Left TLE patients: correlational analysis. Left middle and inferior frontal activation for verbal fluency correlates with naming scores, characterised by better naming scores in subjects with greater fMRI activation (Display at threshold *p* < 0.001, uncorrected). The correlations at the peak voxel are illustrated on the right. Significant regions are superimposed onto an averaged normalized mean EPI image from 30 healthy controls, 15 patients with left and 15 patients with right HS.

Correlation of naming ability with verbal fluency fMRI activation (Table 2). Voxel-wise correlational analysis:

(a) Healthy controls: McKenna Graded Naming Test Scores were significantly related to fMRI activation for verbal fluency in the left hippocampus in healthy controls (*p* = 0.014, FWE corrected in SVI), characterised by greater hippocampal fMRI activation being correlated with better naming scores (Fig. 1B and C).
(b) Right TLE: In right TLE patients, naming scores covaried significantly with left hippocampal fMRI activation for verbal fluency (*p* = 0.049, FWE corrected in SVI) (Fig. 2A).
  In controls or right TLE patients voxel-wise analysis over the whole brain did not show a significant correlation in any other brain areas other than the hippocampus.
(c) Left TLE: In left TLE patients there was no significant correlation between naming scores and left hippocampal fMRI activation for verbal fluency. There was, however, a significant positive correlation in the left middle (*p* = 0.008) and inferior frontal gyri (*p* = 0.024, FWE corrected in a SVI using a sphere of 20 mm diameter centred on the peak activation in the middle (−46/2/54) and inferior (−60/18/18) frontal gyri) (Fig. 2B). There was also a positive correlation in the right middle and inferior frontal gyri, which did not reach statistical significance.

Correlation of verbal fluency performance outside the scanner with verbal fluency fMRI activation.  

Voxel-wise correlational analysis:

Using the same correlational analysis and the same thresholds as for the correlations with naming, verbal fluency outside the scanner was significantly related to fMRI activation for verbal fluency in the left inferior frontal gyrus (*p* = 0.002, FWE corrected in a SVI using a sphere of 20 mm diameter centred on the peak activation in the inferior frontal gyrus (−60/12/24)) in left TLE patients, while there was no significant correlation for controls and right TLE patients. In controls, left and right TLE patients no significant correlation was seen in the hippocampi.

## Discussion

We assessed the integrity of a fronto-temporal language network by including naming and verbal fluency scores as regressors in a between subjects (random effects) analysis of verbal fluency activation. By correlating activation obtained during a verbal fluency fMRI task with out of scanner naming ability we identified brain areas where activation increased in association with improved verbal fluency performance, which is dependant, in part, on recall of names of items. In this way we showed that left hippocampal fMRI activation in a verbal fluency task was associated with object naming proficiency in healthy controls and right TLE patients, highlighting the role of the hippocampus in naming ability, while verbal fluency was shown to be more dependent on frontal structures.

We included a group of age and gender matched control subjects to identify brain areas activated by the task in patients, but not controls. In this way we demonstrated recruitment of specific language areas required for naming involving the left middle and inferior frontal gyri in patients with left TLE.

Verbal fluency tasks usually activate frontal areas, but strongly depend on the functional integrity of a network of language areas, including the dominant inferior frontal lobe, dorsolateral prefrontal cortex, mesio-temporal and parietal lobe (Friedman et al., 1998; Phelps et al., 1997; Pihlajamaki et al., 2000). In keeping with previous studies, in healthy controls our fMRI group results demonstrated activation in these areas while the main effect of verbal fluency relative to fixation was less prominent in the mesio-temporal structures. However, by introducing the covariate of naming ability, we found exclusive hippocampal activation in subjects with a presumed normally functioning hippocampus. While we found a similar, albeit less significant correlation in the left hippocampus in right TLE patients, left TLE patients showed a different pattern, with weaker performance on the graded naming test, and which was associated with significant activation in the left middle and inferior frontal gyri, but not in the mesio-temporal structures.

Clinical evidence for hippocampal involvement in language function is provided by patients with circumscribed focal lesions in the speech-dominant hippocampus showing impaired naming abilities and also by TLE patients, who frequently develop specific language impairment such as difficulties with naming rather than verbal fluency after dominant anterior temporal lobe resections (Powell et al., 2008; Sabsevitz et al., 2003).

The specific role of the hippocampus in episodic memory is well documented (Detre et al., 1998; Dupont et al., 2000; Gaillard, 2004; Powell et al., 2005; Richardson et al., 2004; Squire and Zola-Morgan, 1991). An important aspect highlighting the relationship between memory and language functioning was demonstrated by Binder et al. who found that evaluation of preoperative language dominance as assessed by fMRI was useful in identifying patients at high risk of verbal memory impairment after temporal lobe epilepsy surgery (Binder et al., 2008). Earlier studies also emphasized the importance of the hippocampus in associative processing (Vandenberghe et al., 1996). Our findings suggest that the left hippocampus is engaged in effective word retrieval in healthy subjects and right TLE patients providing further support for the hypothesis that the hippocampus plays a role in retrieving lexically and semantically associated words (Bartha et al., 2003, 2005; Pihlajamaki et al., 2000). In patients with left TLE and HS object naming weakness was paralleled by left hippocampal dis-engagment. A positive correlation in the left and to a lesser extent also in the right middle and inferior frontal gyri suggests compensatory strategies using less functionally developed regions in the frontal lobe supporting naming function. These areas are recruited when the dominant hippocampus is disabled as is the case in hippocampal sclerosis. Naming performance difficulties in left TLE patients might then be explained by the need to rely on a functionally less specialised, compensatory network in the frontal lobe. A similar process has previously been reported for episodic memory in left TLE (Dupont et al., 2000). The underlying mechanisms may either be the pathology (hippocampal sclerosis) or the ongoing epileptic activity and propagation involving temporal and frontal regions, disrupting normal functioning of this part of the expressive language network.

Our study has some limitations:

(1) Our results may be influenced by the effect of volume averaging on the extent and magnitude of hippocampal signal, given that the left TLE patients all had HS.
(2) The verbal fluency task during fMRI was carried out covertly, so that performance was not directly measured in the scanner. We used performance during out of scanner neuropsychological testing as an approximate measure of general motivation and task adherence. In addition all patients underwent one practice run outside the scanner with a different letter than the ones used during the scanning process in order to ensure adequate understanding of the task. Performance differences between controls and patients can simply explain differences in fMRI activations. We have overcome this confound by correlating fMRI activations on a verbal fluency task with a behavioural measure of interest, naming ability. Performance on letter fluency outside the scanner did not correlate with hippocampal activation on verbal fluency inside the scanner suggesting that hippocampal activation predicts naming more than verbal fluency. The Graded Naming test is a more pure measure of naming while the out of scanner verbal fluency task in addition draws upon strategy formation and other executive processes.
(3) We only looked at indicators of expressive language skills by combining a verbal fluency fMRI task which was part of our standard presurgical language fMRI assessment with out of scanner verbal fluency and naming performance, which is of great clinical relevance in patients with TLE especially following ATLR. The verbal fluency task used required recollection of words and was associated with hippocampal activation. However, for future studies it will be favourable to design suitable fMRI paradigms to assess naming function directly in the scanner and to examine receptive language function in more detail.
(4) Early age of epilepsy onset is known to influence reorganisation of language function in TLE. As our patient sample for this study was restricted to patients with left language dominance in order to study language reorganisation in a “methodologically idealized” patient population and as atypical dominance is highly related to an early seizure onset we were not able to study effects of age of seizure onset in this particular study. Within our restricted patient sample there was no relationship between age of epilepsy onset and naming performance. In a next step it would be interesting to compare the extent of hippocampal activation/correlation in patients with pure left language dominance versus those with atypical language representation.

In conclusion, our results demonstrate the importance of the dominant hippocampus for naming function in healthy controls and patients with TLE. Additionally in left TLE patients we demonstrated that decreased naming ability was most likely due to disruption of the hippocampal system either via pathology or epileptic activity and the reliance on functionally less developed frontal lobe networks. Further studies using activation protocols which reliably provoke fMRI activation in these areas will be needed to evaluate the neural pathways in more detail which will then lead to a better understanding of the underlying neurobiological substrate. Prospective follow-up studies are underway to evaluate whether simple fMRI language tasks performed preoperatively may be useful as additional clinical tools to predict clinically relevant naming decline after surgery to the anterior temporal lobe.

## Conflict of interest

None of the authors has any conflict of interest to disclose.

## Figures and Tables

**Table 1:** FMRI activation peaks for the main effects of verbal fluency.

Subjects	*Z*-score	Cluster size	Df, corrected *p*-value (FWE)	Coordinates (*x*, *y*, *z*) in MNI space	Anatomical locations of maxima
Controls	Infinity	12,258	85, *p* < 0.0001	−44, 22, 24	Left middle frontal gyrus
	Infinity	12,258	*p* < 0.0001	−30, 24, 0	Left inferior frontal gyrus
	6.37	499	*p* < 0.0001	32, 22, 0	Right inferior frontal gyrus
	3.88	75	–	−28, −20, −10	Left hippocampus
Left TLE	Infinity	13,124	85, *p* < 0.0001	−50, 8, 20	Left middle frontal gyrus
	Infinity	13,124	*p* < 0.0001	−32, 22, 0	Left inferior frontal gyrus
	6.45	593	*p* < 0.0001	32, 24, 0	Right inferior frontal gyrus
	5.20	59	–	−30, −24, −8	Left hippocampus
Right TLE	Infinity	8320	85, *p* < 0.0001	−50, 10, 22	Left middle frontal gyrus
	7.28	8320	*p* < 0.0001	−32, 22, 0	Left inferior frontal gyrus
	5.8	772	*p* < 0.0001	32, 22, 2	Right inferior frontal gyrus
	2.43	22	–	−30, −26, −6	Left hippocampus
*Group comparisons between patients and controls for the main effects of verbal fluency*					
Left TLE < controls	4.19	47	85, *p* < 0.0001 (uncorrected)	−44, 26, 26	Left middle frontal gyrus
Right TLE < controls	4.48	127	85, *p* < 0.0001 (uncorrected)	−44, 24, 26	Left middle frontal gyrus
Left TLE versus right TLE	ns	–	ns	–	–

MNI space, coordinates related to a standard brain defined by the Montreal Neurological Institute (MNI); ns, not significant; TLE, temporal lobe epilepsy.

**Table 2:** Correlation of McKenna graded naming scores with verbal fluency fMRI activation.

Subjects	*Z*-score	Cluster size	Df, corrected *p*-value (FWE corrected in SVI)	Coordinates (*x*, *y*, *z*) in MNI space	Anatomical region
Controls	2.90	76	78, *p* = 0.014	−24, −20, −18	Left hippocampus
Right TLE	2.38	62	78, *p* = 0.049	−26, −22, −14	Left hippocampus
Left TLE	–	–	ns	–	Left hippocampus
Left TLE	3.54	134	78, *p* = 0.008	−46, 4, 54	Left middle frontal gyrus
Left TLE	3.17	80	*p* = 0.024	−60, 18, 10	Left inferior frontal gyrus

MNI space, coordinates related to a standard brain defined by the Montreal Neurological Institute (MNI); ns, not significant; SVI, small volume of interest; TLE, temporal lobe epilepsy.
